# Pre- and/or Postharvest Silicon Application Prolongs the Vase Life and Enhances the Quality of Cut Peony (*Paeonia lactiflora* Pall.) Flowers

**DOI:** 10.3390/plants10081742

**Published:** 2021-08-23

**Authors:** Jinnan Song, Yali Li, Jiangtao Hu, Jaehyeok Lee, Byoung Ryong Jeong

**Affiliations:** 1 Department of Horticulture, Division of Applied Life Science (BK4 Plus Program), Graduate School of Gyeongsang National University, Jinju 52828, Korea; Jinnansong93@gmail.com (J.S.); lyl016107@126.com (Y.L.); jiangtaoh@yahoo.com (J.H.); 2 Department of Horticulture, College of Agriculture and Life Science, Gyeongsang National University, Jinju 52828, Korea; meatspinang@naver.com; 3 Institute of Agriculture and Life Science, Gyeongsang National University, Jinju 52828, Korea; 4 Research Institute of Life Science, Gyeongsang National University, Jinju 52828, Korea

**Keywords:** holding solution, preservatives, flower stage, antioxidant enzymes, stem strength

## Abstract

Peony is an important ornamental plant and has become increasingly popular for cut flower cultivation. However, a short vase life and frequent poor vase quality severely restrict its market value. The study described herein was conducted to investigate the effects of silicon application on the vase life and quality of two cut peony (*Paeonia lactiflora* Pall.) cultivars, ‘Taebaek’ and ‘Euiseong’. For pre- and/or postharvest silicon application, four experimental groups based on treatments were designed. With silicon treatment, the relevant growth attributes, including the shoot and leaf lengths, stem and bud diameters as well as the leaf width were all remarkably increased. In the postharvest storage, the addition of silicon to the holding solution in the vase was able to significantly extend vase life, delay fresh weight decrease, and improve vase quality, as characterized by the antioxidant enzyme activities and mechanical stem strength. Taken together, silicon application, regardless of the approach, was able to effectively prolong the vase life and enhance the quality of cut peony flowers.

## 1. Introduction

*Paeonia lactiflora* Pall., also known as herbaceous peony, is distributed and cultivated worldwide. It is a highly decorative plant species, owing to its abundant flowers of bright graceful colors and elegant shapes [1]. These characteristics have made it an extremely popular ornamental plant in the global flower market in recent years [2]. This popular plant species with numerous cultivars, featuring lush and often fragrant flowers, are considered suitable for cut flowers. For instance, ‘Taebaek’ and ‘Mulsurae’ have been characterized and developed for commercial production in South Korea [3]. However, a seasonal nature that influences early petal drop and a very limited vase life somewhat threaten its quality and use [4,5]. Postharvest performance is particularly crucial for the cut flower industry, and therefore, addressing this problem is naturally beneficial for the availability and flexibility of the cut peony market. Therefore, it appears that developing effective procedures or approaches capable of extending the vase life while maintaining great flower quality during the postharvest phase is recommended.

To an extent, the plant fresh weight is a direct indicator of a plant’s water content. Dehydration and wilting are two major factors that affect flower opening and senescence by determining the quality and vase life of cut flowers as well as the plant fresh weight. These two physiological changes are caused either by a bacterial block to the plant water uptake or air embolism in the plant stem [6].

Plenty of treatments/techniques to prolong the shelf life of cut flowers have been documented with varying degrees of success. The storage air temperature and humidity are considered very crucial to the quality of postharvest plants; a temperature in the 4–10 °C range is often considered feasible [7]; a relatively low-humidity air storage environment also helps to maintain the vase life of cut flowers well [8]. An appropriate storage air temperature and humidity can only be accomplished using a large equipment or system, unfortunately, which is costly and labor-intensive. The addition of chemical preservatives to the holding solution remains the most common method for extending vase life. Commercial floral preservatives contain a diverse array of chemicals, ranging from carbohydrates, germicides, plant growth regulators (PGRs), surfactants, and mineral compounds, which function to supply energy, control bacterial growth, and improve water uptake [6,9,10]. Most chemical preservatives, such as cobalt sulphate (CoSO_4_), aluminum sulphate (Al_2_(SO_4_)_3_), and silver nitrate (AgNO_3_), are deleterious to humans even though they do an excellent job of enhancing the vase life of flowers [11].

Silicon (Si) is a non-toxic, useful, and abundant element that participates in a wide range of plant activities [12]. Taking plant physiology as an example, Si can reinforce the photosynthesis at the cost of lower transpiration, thus benefiting the nitrogen metabolism and carbon accumulation. Silicon is absorbed and forms a double silicate layer on the leaf epidermis, allowing better leaf architecture and greater light assimilate capacity [13]. Additionally, the Si uptake and transport in the lateral roots are accomplished via the Si accumulators, but in shoots, they are regulated by the transpiration rate [14]. One of the most beneficial roles of Si that has been demonstrated is that it helps plants manage biotic and abiotic stresses [15]. Moreover, many studies have suggested that Si plays an important role in increasing the rigidity of the cell walls of plants, leading to a more erect plant [16]. In addition, researchers have found that Si can stimulate the plant defense system against the infection of *P. ultimum*, which makes plants prone to damping off [17]. Equally as important, Si has a great potential in mitigating the symptoms caused by *Fusarium* wilt, which is believed to destroy water translocation, inducing drooping foliage, leaf chlorosis, and other wilt symptoms [18].

Despite these beneficial characteristics, little research has been conducted to date that assess how Si affects the cut flower quality and vase life of peony. In this study, the effects of pre- and/or postharvest Si treatments on the cut flower quality and vase life in two peony cultivars were analyzed.

## 2. Materials and Methods

### 2.1. Plant Materials and Growth Conditions

For the following experiments, two herbaceous peony (*Paeonia lactiflora* Pall.) cultivars, ‘Taebaek’ and ‘Euiseong’, were selected as the experimental material and were grown under field conditions at Gyeongsang National University (35°90′ N, 128°06′ E, Jinju, Gyeongsangnam-do, Korea) during the 2021 growing season.

### 2.2. Silicon Treatments and Experimental Design

A single silicon application treatment was performed via soil dressing with 5 granular grams of the commercial silicon fertilizer ‘Keunson’ (equivalent to 0.75 g of pure Na_2_SiO_3_) (Saturn Bio Tech Co., Ltd., Gangwon-do, Korea). The plants without any treatments were regarded as the control.

Weeks later, the stems were cut and trimmed to 55 cm with the three topmost leaves remaining when the flowers entered the ‘marble-like’ stage, featuring hard and tight buds, with no visible signs of disease, pests, or mechanical flaws [19]. After cutting, they were immediately transferred to vases with the different holding solutions in a refrigerated showcase (Refrigeration Plus LLC, Colorado Springs, CO, USA), housing a mixed set of white light LEDs and a constant temperature of 20 °C. The holding solutions were distilled water and Si solution, which was determined to be 75 mg·L^−1^, which is in accordance with our lab’s pioneer publication [20]. From the samples, three stems with flowers were individually labeled, placed in a vase, and set as a single replicate. This design with three biological replicates was performed to test the effects of the holding solution on the vase life and quality of the cut flowers.

### 2.3. Growth Attribute Measurements

The growth attributes of the peony were investigated during the harvest, which included the shoot length, leaf length, stem diameter, leaf width, and bud diameter to check if Si positively affected plant growth and development. After being weighed, some fresh leaves were dried using an air-forced oven (FO-450M, Jeio Technology Co. Ltd., Daejeon, Korea) at 70 °C to determine the Si content. The stem and bud diameters were measured using a Vernier caliper (CD-20CPX, Mitutoyo Korea Co., Gunpo, Korea).

### 2.4. Determination of the Si Contents

The method for determining the Si content was a slightly modified version of the protocol found in Zhang’s study [21]. Dried samples were ground into a fine powder, and 0.25 g of the samples were ashed in a Nabertherm muffle furnace (Model LV 5/11/B180, Lilienthal, Bremen, Germany) for more than 2 h at 525 °C. Afterward, the ash was mixed with 5 mL 25% HCl and was then adjusted to 30 mL with 10 mL and 15 mL of deionized water at ambient temperature and 50 °C, respectively. Finally, the Si contents in these solutions were determined three times using an inductively coupled plasma (ICP) spectrometer (Optima 4300DV/5300DV, Perkin Elmer, Germany).

### 2.5. Definitions of Postharvest Flower Opening Stages for ‘Taebaek’ and ‘Euiseong’

**Figure 1:**
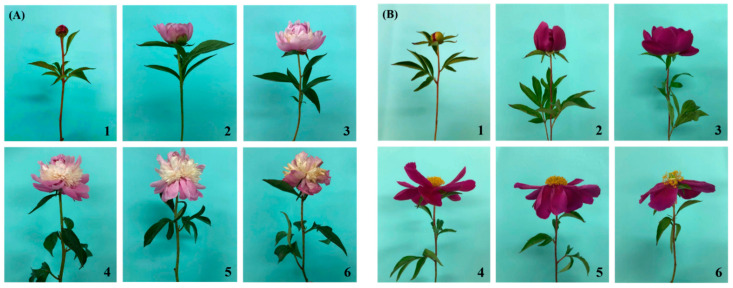
Cut peony flowers of (**A**) ‘Taebaek’ and (**B**) ‘Euiseong’ are divided into six stages according to their postharvest status. Stage 1 ‘Pre-opening’ or ‘marble-like’, Stage 2 ‘Initial-opening’, Stage 3 ‘Half-opening’, Stage 4 ‘ Full-opening’, Stage 5 ‘Petal-wilting’, and Stage 6 ‘Stamen or pistil-wilting’.

The postharvest flower opening stage was evaluated as soon as the cut flowers were placed in the holding solutions. The vase life of the postharvest cut peony flowers could be defined by summing the number of days from harvest to the initial wilting and/or petals falling [22]. The flower opening stages after harvest were markedly classified into six phases based on their changes in appearance (Figure 1). Stage 1 displayed a relatively soft bud and was ready for ‘pre-opening’. Stage 2 was defined as an ‘initial-opening’ condition, where the petals can be readily observed. Stage 3 occurred when the buds were near or past ‘half-opening’. Stage 4 was determined when all of the bud body completely blossomed, which was designated as ‘full-opening’. Stages 5 and Stage 6 were defined as ‘wilting’ accompanied by the petals rolling up and/or falling as well as the further decay of the stamen or pistil.

### 2.6. Measurements of Flowering Stem Fresh Weight during the Vase Life

The individual flowering stems were taken out of the holding solutions daily, surface-blotted with absorbent paper, and subjected to fresh weight measurements. Then, the fresh weight loss per stem was recorded.

### 2.7. Quantification of the Antioxidant Enzyme Activities

The oxidative stresses of the postharvest cut flowers were analyzed when they entered the wilting stage. Fresh petals were collected, immediately immersed in liquid nitrogen, and ground into a fine powder over an ice bath. A total of 100 mg of the powder was homogenized in 50 mM of PBS (1 mM EDTA, 1 mM polyvinylpyrolidone, and 0.05% triton-X, pH = 7.0). This mixture was then centrifuged (13,000 rpm, 4 °C, 20 min) to obtain the supernatant that would be used afterward for total protein estimation and antioxidant enzyme activity assay. The total protein estimations were conducted using Bradford’s reagent [23]. The antioxidant enzymes, including peroxidase (POD), catalase (CAT), superoxide dismutase (SOD), and ascorbate peroxidase (APX), were spectrophotometrically measured following the protocol described by Manivannan et al. [24].

### 2.8. Stem Strength Determination

The stem strengths tested herein are represented by means of the modulus of elasticity (MOE, MPa) and the modulus of rupture (MOR, MPa) when the cut flowers entered the wilting stage. The test specimens were first taken from the intact stem and were then prepared with end faces, which are planar and perpendicular to the loading axis [25]. The MOE and MOR of the neck and base parts were measured using a universal materials testing apparatus (Instron 5965, Instron Corp., Norwood, MA, USA), and calculated according to equations in Onoda’s publication [26].

### 2.9. Statistical Analysis

Statistical analyses were performed using the SAS statistical software (SAS 8.2 Inst., Cary, NC, USA). Data from analysis of variance (ANOVA) and Duncan’s multiple range tests were considered significant at a probability (*p*) equal to 0.05. Fisher’s least significant difference test was used for the *F*-test between treatments. All of the measurements were conducted with no less than three biological replicates.

## 3. Results

### 3.1. Growth and Development of Peony Plants as Affected by Si

The growth attributes of pre-harvest peony, including the shoot length, stem diameter, leaf length, leaf width, and bud diameter, were significantly affected by Si application. As shown in Table 1, all of the growth attributes listed here increased when the plants were subjected to the Si treatments, regardless of the cultivar. For example, relative to the control group (water treatment), the shoot length and leaf length of ‘Taebaek’ improved by 12.53% and 23.38%, respectively; similarly, the same attributes in ‘Euiseong’ increased by 17.04% and 18.87%. Concerning the differences in the attributes of the Si-treated groups and the control (water treatment), notably, ‘Taebaek’ displayed much more prominent increases (34.04% and 20.71%), whereas ‘Euiseong’ displayed relatively milder changes (12.18% and 5.56%) (Table 1). The *F*-test data simultaneously exhibited the highly significant positive influences of the Si applications. It was also demonstrated that the magnitude of the effects of silicon applications on the stem and bud diameters were strongly dependent on the cultivar.

### 3.2. Si Contents in the Harvested Peony

**Figure 2:**
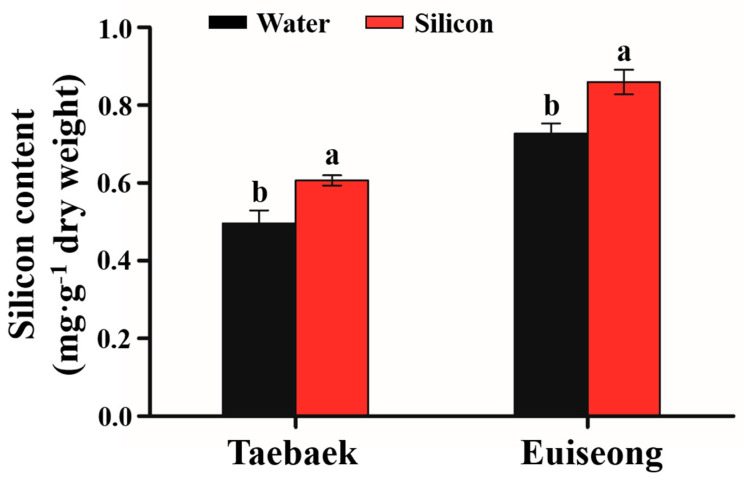
The silicon content in peonies (**a**) ‘Taebaek’ and (**b**) ‘Euiseong’ after the water treatments (Black) and silicon treatments (Red). Values are expressed as the mean ± SD of *n* = 3 biological replicates. Error bars represent standard deviations of the means. Different lowercase letters indicate significant differences according to Duncan’s multiple range test at *p* = 0.05.

The leaves of peonies grown in field conditions (water treatment) and those subjected to the silicon fertilizer ‘Keunson’ (silicon treatment) for many weeks were selected and sampled before harvest. Then, the silicon uptake by the cultivars was investigated, and the silicon content was expressed as milligrams per gram of the dry leaf weight. As shown in Figure 2, the applications of ‘Keunson’ on ‘Taebaek’ and ‘Euiseong’ enhanced the silicon content by 22.30% and 18.24%, respectively, compared to that of the control. However, neither the water treatment nor the silicon treatment on ‘Taebaek’ enhanced the Si content to be higher than that of ‘Euiseong’.

### 3.3. Vase Life of Postharvest Peony

**Figure 3:**
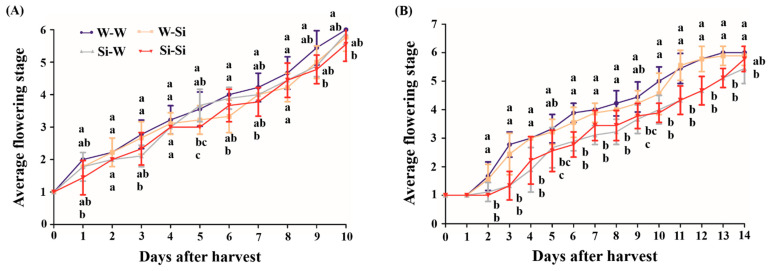
The effects of pre- and/or postharvest silicon treatments on the vase life of cut peony flowers of (**A**) ‘Taebaek’ and (**B**) ‘Euiseong’. ‘W-W’ refers to water treatment with both pre- and postharvest peony plants; ‘W-Si’ refers to water and silicon treatment on pre-harvest and postharvest peony plants, respectively; ‘Si-W’ refers to silicon and water treatments on pre-harvest and postharvest peony plants, respectively; ‘Si-Si’ refers to silicon treatments on both pre- and postharvest peony plants. Values are expressed as means ± SD of *n* = 9 biological replicates. Error bars represent the standard deviations of the means. Different lowercase letters indicate significant differences according to Duncan’s multiple range test at *p* = 0.05. Note that ‘W-W’, ‘W-Si’, ‘Si-W’, and ‘Si-Si’ mentioned henceforth carry the same meaning as used here.

The vase life of each peony cultivar was determined by individually collecting the flowering stage data of nine peony flowers, represented in three replicates for each treatment. Comparing Figure 3A with Figure 3B reveals that the vase life of cut ‘Taebaek’ and ‘Euiseong’ peony flowers displayed some remarkable differences, where the ‘Euiseong’ flowers had four more days of vase life compared to the ‘Taebaek’ flowers. More broadly, the Si treatments were able to retard and sustain one to two days of vase life in ‘Taebaek’ and three to four days in ‘Euiseong’ during the identical flowering stage.

As displayed in Figure 3, it is noteworthy that the peonies treated with water either in the pre- or postharvest phases (the control: ‘W-W’) showed an earlier flowering stage and a shorter vase life as well as a more susceptible senescence, regardless of the cultivar; the peony plants and flowers subjected to the Si treatment either in the pre- or postharvest phases (‘Si-Si’) possessed the most outstanding results, including not only the vase life but also the speed of flower senescence. Peony plants in the ‘W-W’ group developed approximately one flowering stage ahead of those in the ‘Si-Si’ groups, and a flowering stage growth curve of the peony plants subjected to the Si treatments in pre-harvest and water treatments in postharvest (‘Si-W’) that was similar to that of the ‘Si-Si’ groups was observed. The ‘W-Si’ group displayed a flower stage growth curve which was reduced by about half of a flowering stage compared to the curve for the plants in the ‘W-W’ groups. The flowering stage was significantly extended by 1/2-1 flowering stage by the Si treatments (Figure 3A). From days 9 to 13, the flowering stages of peony plants in the ‘Si-W’ groups overlapped with those in the ‘Si-Si’ groups, while transitioning from Stage 3 to Stage 4; at this moment, the plants in the ‘W-W’ groups had an analogous flowering stage growth curve to that of those in the ‘W-Si’ groups, which transitioned from Stage 5 to Stage 6 (Figure 3B).

### 3.4. Fresh Weight of Postharvest Peony

**Figure 4:**
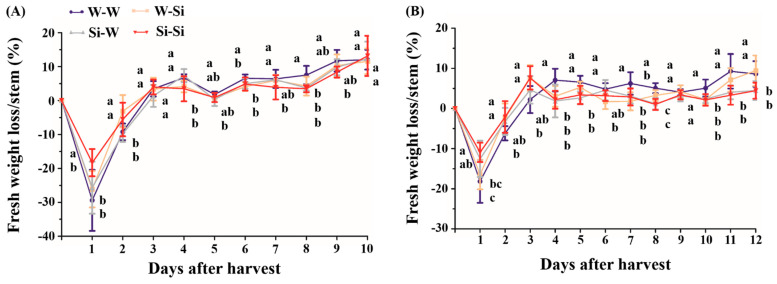
The effects of pre- and/or postharvest silicon treatments on the fresh weight of cut peony flowers from (**A**) ‘Taebaek’ and (**B**) ‘Euiseong’ during vase life. Fresh weight loss/stem is analyzed by calculating the average percentage of fresh weight loss against harvest days. Values are expressed as the means ± SD of *n* = 9 biological replicates. Error bars represent the standard deviations of the means. Different lowercase letters indicate significant differences according to Duncan’s multiple range test at *p* = 0.05.

Identical to the flower stage assay above, fresh weight measurements were simultaneously performed on the individual cut peony flowers during the vase life test. All of the cut peony flowers, regardless of the cultivar and treatment, suffered a sharp water uptake on day 1 of the vase life, which then rapidly decreased on day 2. Both ‘Taebaek’ and ‘Euiseong’ gradually lost water from day 3 onward (Figure 4).

However, the water uptake rate varies according to the cultivar and treatment. The greatest water uptake amount in ‘Taebaek’ was determined to be 15–30%, while this value was 10–20% in ‘Euiseong’ (day 1). Concomitantly, the water uptake rate of cut flowers in the ‘Si-Si’ groups was the lowest (18% and 10% for ‘Taebaek’ and ‘Euiseong’, respectively), while the highest water uptake rate was 29% and 18% for ‘Taebaek’ and ‘Euiseong’, respectively, as displayed in the ‘W-W’ groups (Figure 4).

Still, a major difference in the fresh weight loss/stem can be observed according to the cultivar or treatment. The cut peony flowers in the ‘W-W’ groups consistently exhibited the highest fresh weight loss out of all the considered groups. The biggest difference appeared, as expected, between the ‘W-W’ groups and ‘Si-Si’ groups, which were determined to be as high as 5~6% in ‘Euiseong’ (day 4 and day 11) and 4% in ‘Taebaek’ (day 8). In addition, there was also a minor decrease in the fresh weight loss in the ‘W-Si’ and ‘Si-W’ groups compared to the ‘W-W’ groups, such as ‘Taebaek’ and ‘Euiseong’ on day 9, at 1.5%, 1.9% and 0.16%, 0.9%, respectively (Figure 4).

### 3.5. Antioxidant Enzyme Activities

**Figure 5:**
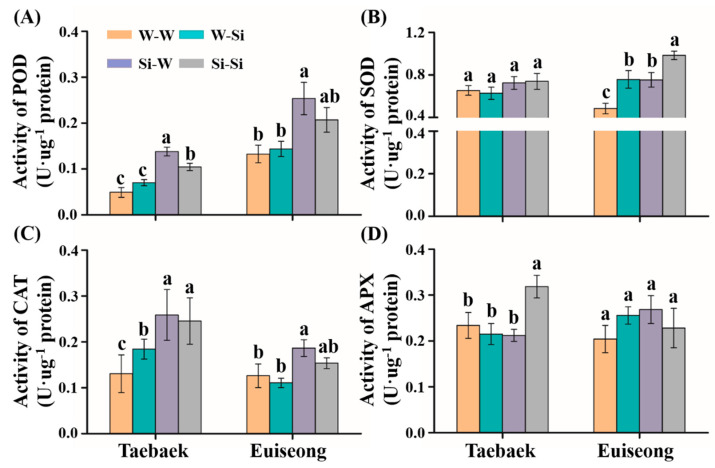
Activities of (**A**) peroxidase (POD); (**B**) superoxide dismutase (SOD); (**C**) catalase (CAT); and (**D**) ascorbate peroxidase (APX) in postharvest cut peony flowers of ‘Taebaek’ and ‘Euiseong’ at the wilting stages. Values are expressed as the means ± SD of *n* = 9 biological replicates. Error bars represent the standard deviations of the means. Different lowercase letters indicate significant differences according to Duncan’s multiple range test at *p* = 0.05.

The oxidative protective system was triggered when the plants suffered from abiotic stresses. The antioxidant enzyme activities regarding POD, SOD, CAT, and APX were quantified in the fallen petals. On average, it is noteworthy that all of the four protective enzymes displayed elevated activities relative to the ‘W-W’ groups, among which pre-harvest silicon treatments (‘Si-W’ and ‘Si-Si’) were superior to either the postharvest silicon treatment (‘W-Si’) or the control (‘W-W’) on promoting the POD and CAT activities regardless of the cultivar (Figure 5A,C). However, the activity of SOD in ‘Taebaek’ subjected to the pre-harvest silicon treatments (‘Si-W’ and ‘Si-Si’) was only slightly enhanced compared to that of the control (‘W-W’), a similar trend was observed regarding the activity of APX in ‘Euiseong’ (Figure 5B,D). Furthermore, the greatest differences in ‘Taebaek’ and ‘Euiseong’ between the groups were observed in the POD activity, which dramatically increased by 64.5% and 47.4%, respectively (Figure 5A).

### 3.6. MOE and MOR of Postharvest Peony

There were two different mechanical properties, the modulus of elasticity (MOE) and the modulus of rupture (MOR) of intact stems, that were immediately examined on the last day after harvest. A wide range of variations was found in their values. Overall, it is noteworthy that the Si applications significantly increased the MOE value and MOR value no matter the cultivar (Table 2).

Regarding the MOE value for the neck part in ‘Taebaek’, more rapid increases of 92.4%, 79.5%, and 59.4% were detected in the ‘Si-Si’, ‘Si-W’, and ‘W-Si’ groups, respectively, compared to the control ‘W-W’ groups; moreover, similar trends in ‘Euiseong’ were obtained, with relative increases at 94.8%, 68.7%, and 21.1% in the ‘Si-Si’, ‘W-Si’, and ‘Si-W’ groups, respectively, compared to the control ‘W-W’ groups. Meanwhile, such comparisons were also conducted for the base part, where the ‘Si-W’ group showed a 1.6-fold higher MOE than that of the ‘W-W’ group in ‘Taebaek’. The greatest such difference in ‘Euiseong’ was the 1.2-fold increase of the MOE of the ‘W-Si’ group compared to that of the ‘W-W’ group (Table 2 ‘MOE part’).

The effects of Si on the MOR value at the neck part were more pronounced compared to those at the base part, regardless of the cultivar. No differences were observed regarding the MOR at the base part among the different treatment groups in ‘Taebaek’ nor ‘Euiseong’. For instance, the MOR of the ‘W-W’ group was even higher than that of the ‘W-Si’ and ‘Si-W’ groups in ‘Taebaek’ and the ‘Si-W’ group in ‘Euiseong’ (Table 2 ‘MOR part’).

## 4. Discussion

Although Si is still not considered as an essential element for higher quality plants, the evidence demonstrating its beneficial influences is growing [27]. Certain outstanding roles, such as alleviating the abiotic stresses, reducing wilting, and upgrading the mechanical strength, have been confirmed [28,29,30], and these characteristics are, accordingly, associated with the longevity and quality of cut flowers. Strategies for the modifications of the ingredients in holding solutions were believed to be highly feasible for prolonging vase life. Traditionally, plenty of cut flower preservatives have been tested, which are effective but toxic and costly in large-scale applications [31]. Furthermore, little research has focused on the form and rate of Si used in spite of some of the positive aspects of Si applications that have been reported in ornamental species [32,33]. For instance, bulk attempts that have been too large in size have been conducted by supplementing chemical silicon for postharvest plants, resulting in a shortage of reports regarding silicon use during the pre-harvest process. Therefore, this work was designed, concentrated, and performed to examine the effects of silicon on the vase life of peony via pre- and/or postharvest approaches. It is worthy to note that we minimized the influences of the field environment on the pre-harvest experiment because this research was not only designed as a completely randomized layout, but also had three biological replications. Additionally, plants with consistent growth were selected prior to the treatments, and the silicate fertilizer ‘Keunson’ has quick solubility and great availability. In addition, significant differences in Si content were observed (Figure 2), which meant that the varying growth attributes were promoted by the Si application, leaving little doubt of significant errors induced by the field environment. Additionally, the postharvest experiment was conducted in a controlled environmental regime, which may have negligible influence on the environment. Consequently, in our trials, the peony subjected to both pre-harvest and postharvest silicon treatments (‘Si-Si’) exhibited a prolonged vase life and enhanced plant quality.

Silicon has been well suggested to promote plant quality in many aspects; in particular, considerable regard has been given to it with respect to its plant growth benefits [34]. Our findings revealed that applications of ‘Keunson’ significantly upgraded plant growth and development, involving the shoot length, leaf length and width, and stem and bud diameters, irrespective of the cultivars (Table 1). This was in line with our lab’s previous study and once gain confirmed the promising role of Si [35]. More importantly, the *F*-test data displayed a robust interaction between the Si applications and the diameters (stem and bud), where a vigorous stem was less susceptible to wilting during vase life. From a practical perspective, a pre-harvest Si application may be superior for controlling vase life.

The postharvest flower stage is believed to be a vital indicator of vase life [36]. In our trials, the flower stages of ‘Taebaek’ and ‘Euiseong’ were distinctively divided into six periods based on the flower opening appearances (Figure 1). This standard may serve other species as well. After harvest, we subsequently grouped and placed them into two well-prepared holding solutions to examine the functions of Si as a cut flower preservative (Figure 3). Moreover, a pre-harvest silicon treatment was proven to be superior to a pre-harvest water treatment due to the better performance of the ‘Si-W’ groups relative to the ‘W-Si’ groups (Figure 3). Moreover, vase life varies among species, and even varies between cultivars, which commonly ranges from 3 to 15 days [37,38,39]. In our case, ‘Euiseong’ was observed to possess a vase life that was four days longer than that of ‘Taebaek’ (Figure 3), which is probably caused by the reasons mentioned above.

Likewise, apart from the postharvest flower stage, the water uptake and water loss of cut flowers are two major indicators of the vase life [34]. Our data presented in Figure 4 showed a very similar tendency: the peony plants in the ‘Si-Si’ groups performed the best followed by those in the ‘Si-W’ groups. Most likely, as Jaiganesh [40] noted, Si is able to depress excessive water loss and/or limit bacterial development, resulting in a water balance. Additionally, on the first three days after harvest, the peony plants in the ‘W-W’ groups suffered more rapid water uptake and water loss, which indicated a poor water status. Accordingly, the flowering stages and fresh weight indicators were both markedly benefited by the Si applications. In short, Si markedly referred to its roles in prolonging the vase life as well as in maintaining the water status of cut peony flowers. These data may be explained by the fact that transpiration was reduced by the presence of Si in the form of silica in the thickened leaf epidermis [13,14].

To overcome the unfavorable conditions, plants have developed sophisticated acclimatization strategies, such as the ROS (reactive oxygen species) scavenging system [41]. The activity of the protective enzymes involving peroxidase (POD), catalase (CAT), etc., is boosted during plant senescence. Usually, a greater ability to eliminate ROS indicates higher antioxidant enzyme activity. Moreover, Si has been proven to be associated with a delay in plant senescence by means of increasing the active cytokinin concentration in plants [42]. Here, in the entirety of Figure 5, the activities of POD, CAT, SOD, and APX increased in the silicon-treated groups compared to those in the ‘W-W’ groups, which was probably because of the inference that silicon supply stimulated a more active ROS scavenging system, thereby delaying senescence in maintaining vase life. Interestingly, even though the POD and CAT activities in the ‘Si-W’ groups were higher than those in the ‘Si-Si’ group, the positive aspects of Si were still demonstrated in increasing the quality of cut flowers. Here, an extensive deployment of the protective system was triggered by Si regarding the improvement of antioxidant activity. This was probably ascribed to the participation of Si not only in the reinforcement of organelle structures, but also in the maintenance of integrity of membrane of mitochondria and chloroplasts, which regulate the ROS scavenging system [13].

The measurement of mechanical properties, such as the modulus of elasticity (MOE) and the modulus of rupture (MOR), of the plant stem helps in the investigation of the stem strength [43]. Silicon enhanced the lodging resistance and improved the mechanical strength [44,45,46]. In our trials, the MOR values tested in the neck portion and base portion were distinctly higher in the ‘W-Si’, ‘Si-W’, and ‘Si-Si’ groups when compared to those in the ‘W-W’ groups (Table 2), which can clearly be attributed to the pre- and/or postharvest Si applications. Curiously, the MOE values in the base part of ‘Si-W’ always displayed a relatively lower value (Table 2), which was probably caused by an irregular silicon distribution in the plant stems. For similar reasons to those in the above discussion, it is speculated that the enhanced mechanical strength of the stems was dominated by this deposition of Si in the plants, thereby lowering the wilting speed and impeding the penetration of the decaying microorganisms. However, the mechanisms of Si transport and defense behaviors provided by Si in plants are far from being fully understood.

Many studies have been conducted to investigate the positive effects of Si application on enhancing the vase life of cut flowers. However, unfortunately, they failed either to introduce a pre-harvest Si or modify the holding solution [47,48]. Still, little information of Si effects on peony is available so far. We noticed that only Zhao [30] has proposed a relationship between Si application and the mechanical strength of peony inflorescence stems, although that research was presented in a totally different orientation than our study was.

## 5. Conclusions

In conclusion, soil supplementation with Si significantly promoted peony plant growth and development, and the Si was subsequently absorbed and assimilated in the leaves. Afterwards, testing the addition of Si to the holding solution of cut peony flowers showed that Si prolonged vase life, maintained plant fresh weight, and enhanced plant quality concerning the antioxidant enzyme activities and stem mechanical strength. Hence, Si application is strongly recommended not only in peony garden cultivations but also to maintain a high quality of cut flowers. Moreover, as expected, a pre-harvest Si application in combination with a postharvest Si holding solution was the most promising method for maintaining the high quality of cut flowers.

## Figures and Tables

**Table 1:** Growth attributes of two peony cultivars subjected to water and Si treatments.

Cultivar (A)	Treatment (B)	Shoot Length (cm)	Stem Diameter (mm)	Leaf Length (cm)	Leaf Width (cm)	Bud Diameter (mm)
‘Taebaek’	Water	74.13 b ^z^	3.29 b	9.58 b	3.25 b	23.71 b
	Si	83.42 a	4.41 a	11.82 a	4.13 a	28.62 a
‘Euiseong’	Water	69.42 b	3.53 a	8.85 b	3.22 a	26.26 a
	Si	81.25 a	3.96 a	10.52 a	3.53 a	27.72 a
*F*-test	A	* ^y^	NS	**	NS	NS
	B	***	***	***	**	***
	A × B	NS	*	NS	NS	**

^Z^ indicate separation within columns by Duncan’s multiple range test at *p* = 0.05 via different lowercase letters. ^y^ NS, *, **, ***, non-significant at probability (*p*) ≤ 0.05, 0.01, or 0.001, respectively.

**Table 2:** MOE and MOR values of two peony cultivars subjected to different treatments.

(**A**) MOE and MOR values of the peony ‘Taebaek’ subjected to different treatments					
**Treatment**		**Modulus of elasticity** **(MOE, MPa)**		**Modulus of rupture** **(MOR, MPa)**	
**Pre-harvest**	**Postharvest**	**Neck**	**Base**	**Neck**	**Base**
Water	Water	186.0 d ^Z^	183.0 d	8.34 b	12.13 ab
Water	Si	296.5 bc	351.1 c	11.92 ab	12.03 ab
Si	Water	333.8 ab	487.7 ab	12.07 a	11.95 ab
Si	Si	357.9 a	454.6 ab	12.24 a	13.61 a
(**B**) MOE and MOR values of the peony ‘Euiseong’ subjected to different treatments					
**Treatment**		**Modulus of elasticity** **(MOE, MPa)**		**Modulus of rupture** **(MOR, MPa)**	
**Pre-harvest**	**Postharvest**	**Neck**	**Base**	**Neck**	**Base**
Water	Water	112.1 b	152.0 c	7.1 c	11.3 ab
Water	Si	189.1 a	334.8 a	18.6 ab	15.5 a
Si	Water	138.5 b	232.2 ab	11.2 bc	8.8 b
Si	Si	218.4 a	331.1 a	22.1 a	13.1 ab

^Z^ mean separation within columns by Duncan’s multiple range test at *p* = 0.05 via different lowercase letters.

## Data Availability

Data sharing is not applicable to this article.
